# Characterisation of canine *KCNIP4*: A novel gene for cerebellar ataxia identified by whole-genome sequencing two affected Norwegian Buhund dogs

**DOI:** 10.1371/journal.pgen.1008527

**Published:** 2020-01-30

**Authors:** Christopher A. Jenkins, Lajos Kalmar, Kaspar Matiasek, Lorenzo Mari, Kaisa Kyöstilä, Hannes Lohi, Ellen C. Schofield, Cathryn S. Mellersh, Luisa De Risio, Sally L. Ricketts

**Affiliations:** 1 Kennel Club Genetics Centre, Animal Health Trust, Newmarket, Suffolk, United Kingdom; 2 Department of Veterinary Medicine, University of Cambridge, Cambridge, Cambridgeshire, United Kingdom; 3 Section of Clinical & Comparative Neuropathology, Centre for Clinical Veterinary Medicine, Ludwig-Maximilians-Universität Munich, München, Germany; 4 Neurology/Neurosurgery Service, Centre for Small Animal Studies, Animal Health Trust, Newmarket, Suffolk, United Kingdom; 5 Department of Veterinary Biosciences, and Department of Medical and Clinical Genetics, University of Helsinki, Helsinki, Finland; 6 Folkhälsan Research Center, Helsinki, Finland; University of Edinburgh, UNITED KINGDOM

## Abstract

A form of hereditary cerebellar ataxia has recently been described in the Norwegian Buhund dog breed. This study aimed to identify the genetic cause of the disease. Whole-genome sequencing of two Norwegian Buhund siblings diagnosed with progressive cerebellar ataxia was carried out, and sequences compared with 405 whole genome sequences of dogs of other breeds to filter benign common variants. Nine variants predicted to be deleterious segregated among the genomes in concordance with an autosomal recessive mode of inheritance, only one of which segregated within the breed when genotyped in additional Norwegian Buhunds. In total this variant was assessed in 802 whole genome sequences, and genotyped in an additional 505 unaffected dogs (including 146 Buhunds), and only four affected Norwegian Buhunds were homozygous for the variant. The variant identified, a T to C single nucleotide polymorphism (SNP) (NC_006585.3:g.88890674T>C), is predicted to cause a tryptophan to arginine substitution in a highly conserved region of the potassium voltage-gated channel interacting protein KCNIP4. This gene has not been implicated previously in hereditary ataxia in any species. Evaluation of KCNIP4 protein expression through western blot and immunohistochemical analysis using cerebellum tissue of affected and control dogs demonstrated that the mutation causes a dramatic reduction of KCNIP4 protein expression. The expression of alternative *KCNIP4* transcripts within the canine cerebellum, and regional differences in KCNIP4 protein expression, were characterised through RT-PCR and immunohistochemistry respectively. The voltage-gated potassium channel protein KCND3 has previously been implicated in spinocerebellar ataxia, and our findings suggest that the Kv4 channel complex KCNIP accessory subunits also have an essential role in voltage-gated potassium channel function in the cerebellum and should be investigated as potential candidate genes for cerebellar ataxia in future studies in other species.

## Author summary

Hereditary ataxias, which are a group of disorders characterised by incoordination of movement, are typically incurable and there are often no disease-modifying treatments available. Canine hereditary ataxias are a notable group of movement disorders in dogs, and represent well characterised naturally occurring disease models of ataxia that can help improve our understanding of the underlying biology of the disorder in both dogs and humans. We used the whole genome sequences of two affected siblings to investigate the genetic cause of a slowly progressive form of hereditary ataxia in the Norwegian Buhund dog breed, and identified a single base change within the *KCNIP4* gene. We have characterised the expression of *KCNIP4* in the dog, and investigated the effect of the identified mutation. This gene has not previously been implicated in inherited ataxia in any species, and our findings suggest that this and related genes represent potential candidates for ataxia in future studies in other species. Our findings will allow dog breeders to avoid producing affected dogs, reduce the disease allele frequency, and eventually eliminate the disease from the breed, through the use of a DNA test.

## Introduction

Hereditary ataxias are a group of movement disorders, typified by incoordination of gait, limbs, or eyes, primarily caused by inherited dysfunction of the cerebellum and/or its afferent or efferent pathways [1]. In humans autosomal recessive and dominant forms of hereditary ataxia have been reported, in addition to mitochondrial, and, in the case of fragile X tremor-ataxia, X-linked forms. Inherited ataxias are typically not curable and there are often not any disease-modifying treatments available [1, 2].

Multiple examples of hereditary ataxia have been described in purebred dogs [3–9]. Although the specific diseases are often rare, and the genetic mutations can be breed-specific, hereditary ataxia is a key cause of movement disorders in dogs. Canine hereditary ataxia is typically inherited in an autosomal recessive manner, and disease-causing variants have been identified for some breeds [3–12]. Some of the genes implicated in canine ataxia had not previously been associated with disease in humans [12–15], whereas other forms of canine ataxia are caused by variants within the same genes that are associated with well characterised forms of human ataxia [4–6, 16]. Canine hereditary ataxia is a naturally occurring disease model and research into the genetics of ataxia in purebred dogs can help improve the understanding of the underlying molecular mechanisms of human disease.

Hereditary ataxias in humans are classified by mode of inheritance, whereas in dogs, where the underlying genetic basis is often less well defined, have recently been classified based on the clinical signs and the neuronal structures affected [1, 3]. The five classes of canine hereditary ataxia are cerebellar cortical degeneration (CCD), spinocerebellar degeneration, canine multiple system degeneration (CMSD), cerebellar ataxias without significant neurodegeneration, and episodic ataxias [3].

Different genetic approaches have successfully been used to identify the causal mutations for canine hereditary ataxia. Homozygosity mapping, linkage analysis, and targeted resequencing was used to investigate spinocerebellar ataxia in the Italian Spinone, and identified a GAA repeat expansion in *ITPR1*, a calcium channel that regulates intracellular calcium levels [4]. mRNA sequencing and a candidate gene approach was used to identify an 8bp deletion in *SPTBN2* (β-III spectrin, involved in the development of Purkinje cells) causing CCD in Beagles [16]. Genome-wide association studies (GWAS) have been used to identify the causal mutations for CCD in Finnish Hounds (*SEL1L*, targeted degradation of misfolded or unassembled peptides), and Old English Sheepdogs and Gordon Setters (*RAB24*, which has a role in autophagy); and spinocerebellar ataxia in Russell Terrier Group dogs (*CAPN1*, which encodes μ-calpain, a subunit of calcium dependent cysteine protease) [13–15]. Whole genome sequencing of single cases of ataxia has been used more recently to successfully identify causal variants for CCD in the Hungarian Vizsla (*SNX14*, which has a role in the maintenance of neuronal excitability and synaptic transmission, and is associated with cerebellar ataxia in humans) [5] and spinocerebellar ataxia in Russell Group Terriers (*KCNJ10*, a potassium channel gene) [17]. Both of these studies used control genomes, and predicted effect on the protein sequence, to filter common benign variants, and then identified variants within candidate genes for genotyping in additional dogs.

The clinical and histopathological characteristics of hereditary cerebellar ataxia in the Norwegian Buhund have been described previously in a study of four cases presenting with mild and slowly progressive cerebellar ataxia, characterised by a broad base stance and hypermetria in all four limbs, truncal ataxia, and fine head tremors [18]. At referral two dogs (siblings) were aged 12 weeks, and two other affected dogs were aged 16 and 20 weeks. Histopathological analysis of the cerebellum showed minor signs of degeneration and reduced expression of Purkinje cell differentiation markers calbindin-D-28K and ITPR1 in some cerebellar regions [18]. Pedigree analysis suggested an autosomal recessive mode of inheritance. Genome-wide mRNA-sequencing of two affected siblings and subsequent investigation of 20 candidate genes in this dataset did not identify any potentially causal variants for the disease [18].

The present study aimed to identify the underlying genetic cause of ataxia in the Norwegian Buhund breed using whole genome sequencing of the two affected siblings. A mutation within *KCNIP4*, a novel gene for cerebellar ataxia, was identified.

## Results

### Whole genome variant filtering

Whole genome sequences of two ataxia-affected Norwegian Buhund siblings were initially compared with the Boxer reference sequence CanFam3.1 and whole genome sequences of 44 dogs of 29 different breeds (control genomes). Variants identified were filtered to leave only those which were homozygous in both of the affected dogs, but which were homozygous for the reference or an alternative allele in the control dogs. Our hypothesis was that the causal variant is rare and private to the Norwegian Buhund breed. From this, a total of 26,073 segregating variants were identified. Additional filtering by variant effect, leaving only high-effect variants that were predicted to directly affect a protein coding sequence, or disrupt a transcript, reduced the number to 121.

The 121 variants were then further filtered using whole genome sequence variant data from the Dog Biomedical Variant Database Consortium (DBVDC) [19]. The consortium dataset included 361 additional whole genome sequences comprising 96 different pure breeds, three wolves, and seven types of mixed-breed dog. The variants were filtered to leave only those absent in all of the consortium genomes. This left 16 high-effect variants that were homozygous in the two affected dogs and were not present in any of the other whole genome sequences. Seven of the 16 variants were predicted to be tolerated and benign by two variant effect prediction tools; SIFT and PolyPhen-2. The nine remaining variants were taken forward for genotyping in additional Norwegian Buhunds.

### Variant segregation in additional dogs

Each of the nine variants was initially genotyped in 14 additional, unaffected, Buhunds. These included two full siblings of the affected dogs and two obligate carriers which were identified through the pedigree (S1 Fig). Only one of the nine variants segregated as would be expected for an autosomal recessive mode of inheritance: a nonsynonymous SNP in the *KCNIP4* gene (NC_006585.3:g.88890674T>C) (S1 Table). This variant was genotyped in a further 56 Buhunds (archived DNA samples collected between 2008 and 2011) not reported to have ataxia (a total of 70 tested “UK Buhund set 1”). Of these 70 dogs 24 were heterozygous for the *KCNIP4* variant (T/C), and the other 46 were homozygous for the CanFam3.1 reference allele (T/T) (Table 1). None of the unaffected dogs were homozygous for the *KCNIP4* variant. Genotyping unaffected Norwegian Buhunds sampled in 2017 revealed that this contemporary set of 36 UK dogs (“UK Buhund Set 2”) included three heterozygotes, and within 40 dogs sampled in Finland (“Finnish Buhund Set”) one heterozygote was identified (Table 1). Neither of these sample sets included any dogs homozygous for the variant. Two additional Norwegian Buhunds were previously diagnosed with cerebellar ataxia in 1998 and 2002. Genotyping these additional cases, in addition to the two siblings used for whole genome sequencing, confirmed that all four affected dogs were homozygous for the KCNIP4 variant (Table 1).

**Table 1:** Genotypes of Norwegian Buhunds and a multi-breed panel for the *KCNIP4* variant.

	T/T	T/C	C/C	Total
Buhund Cases	0	0	4	4
UK Buhund Set 1	46	24	0	70
UK Buhund Set 2	33	3	0	36
Finnish Buhund Set	39	1	0	40
Multi-breed Panel	359	0	0	359
Total	477	28	4	509

For further validation and to investigate if the variant is confined to the Norwegian Buhund breed a panel of 359 dogs of 122 other breeds was genotyped. The *KCNIP4* variant was not present in this multi-breed panel (Table 1) (S2 Table).

Variant data for additional whole genome sequences subsequently became available after the initial analysis. These additional genomes included 140 in-house genomes and 255 genomes in the DBVDC consortium [19], all of which were homozygous for the reference allele.

In total the *KCNIP4* variant was assessed in 802 whole genome sequences, including dogs of 158 breeds, 13 mixed breed dogs, and 8 wolves (S3 Table), and genotyped in an additional 505 unaffected dogs (including 146 Norwegian Buhunds), and only the four affected Buhunds (two whole genome sequences and two genotyped) were homozygous for the variant. These results demonstrate that the variant segregates with disease and is confined to the Norwegian Buhund breed.

### Bioinformatics tools predict the KCNIP4 variant to be deleterious

The *KCNIP4* variant is a nonsynonymous T/C SNP causing a tryptophan to arginine amino acid change. The nucleotide, and codon within which the *KCNIP4* variant is located, is conserved across 99 species of mammal (UCSC) (S4 Table). Tryptophan is highly conserved at this location, and so is the flanking amino acid sequence in 19 species of mammal (16 primates) (UCSC) (S2 Fig). SIFT predicted the variant to be “deleterious” (SIFT value: 0), and Polyphen-2 “probably damaging” (Polyphen-2 value: 0.99) [20, 21]. The effect of the variant was also assessed using a third tool, Mutation Taster, which predicted the variant to be “disease causing” (probability: 0.99) [22].

### At least five KCNIP4 transcripts with alternative first exons are expressed in canine cerebellar tissue

**Fig 1:**
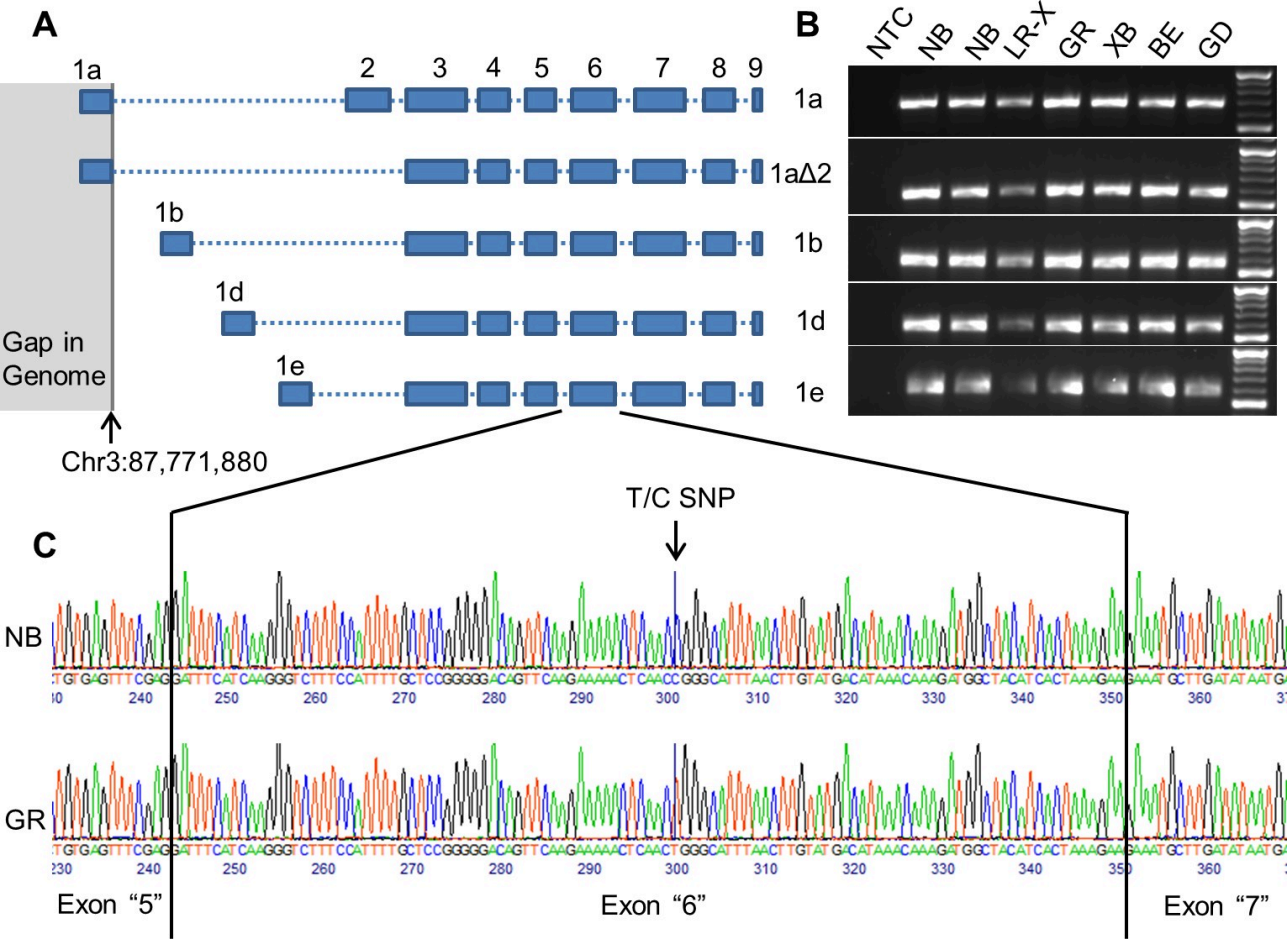
(A) An illustration of the exon composition of the five canine *KCNIP4* transcripts identified using mRNA and whole genome sequencing data and alignment with known human transcripts. KCNIP4-1a and KCNIP4-1aΔ2 share exon 1a as their first exon, whereas all other transcripts have unique first exons. In these five canine transcripts, exon 2 is only present in KCNIP4-1a. Exons 3 to 9 are shared across all five transcripts. Coordinates for the first and second exons of each transcript are in S5 Table. The genomic region containing exons 3 to 9 as labelled in the Figure is Chr3: 88,780,584–88,894,638. The genomic region containing all of the transcripts is Chr3: 87,771,818–88,894,638. (B) RT-PCR of five *KCNIP4* transcripts in canine cerebellum samples for seven dogs. From left to right: no template water control (NTC); two ataxia-affected Norwegian Buhunds (NB); Labrador Retriever cross breed (LR-X); Golden Retriever (GR); Siberian Husky cross breed (XB); Beagle (BE); Great Dane (GD). (C) Sanger sequencing chromatogram demonstrating that exon “6”, containing the mutation, is present in the transcripts expressed in the canine cerebellum. The sequences shown are for KCNIP4-1bΔ2. NB: Norwegian Buhund, GR: Golden Retriever.

There are four NCBI RefSeq *KCNIP4* transcripts, and seven Ensembl *KCNIP4* transcripts, annotated for the canine genome (S5 Table). Two of the canine Ensembl transcripts (ENSCAFT00000060142.1, ENSCAFT00000083618.1) match canine RefSeq transcripts (XM_014112663.2, XM_003434400.4), making a total of nine unique *KCNIP4* transcripts annotated for the canine genome. Three of the NCBI RefSeq canine *KCNIP4* transcripts correspond to human Ensembl transcripts and transcripts reported previously to be expressed in human and mouse cerebellum [23] (S5 Table). The corresponding protein RefSeq for canine transcripts XM_014112663.2, XM_003434400.4, and XM_005618660.3 align to the protein sequences for human transcripts KCNIP4-1bΔ2 (ENST00000382148.7), KCNIP4-1dΔ2 (ENST00000382150.8), and KCNIP4-1eΔ2 (ENST00000382149.9) respectively (S5 Table) (Fig 1A). These three transcripts were confirmed using RNA sequencing data for canine cerebellum from dogs of multiple breeds, including the two affected Norwegian Buhund siblings.

Two other transcripts (KCNIP4-1a and KCNIP4-1aΔ2) which have been reported to be expressed in human and mouse cerebellum do not have corresponding canine RefSeq transcripts [23] (S5 Table). An assessment of the syntenic region for the first exon for these transcripts in CanFam3.1 revealed that there is a gap in the canine genome at this location (CanFam3.1 Chr3:87,771,558–87,771,880) (Fig 1A). When translated into the amino acid sequence, unmapped reads from the RNA sequencing data which were paired to the mapped reads of the flanking exons (exon 2 and exon 3), aligned with the N-terminal protein sequence corresponding to the first exon of human transcripts KCNIP4-1a (ENST00000382152.7), which contains “exon 2”, and KCNIP4-1aΔ2 (ENST00000447367.6), in which “exon 3” is the second exon (Fig 1A) (S5 Table). Reads extending upstream into the gap in the genome from the gap’s 3’ end (chr3:87,771,880) aligned with part of the missing sequence, confirming that this gap in the genome is the location of the unaligned exon. A comparison of the identified transcripts, and the sequence and genomic position of canine Ensembl transcripts ENSCAFT00000090603.1 and ENSCAFT00000090861.1, revealed that they are partial canine transcript annotations for KCNIP4-1a and KCNIP4-1aΔ2, but are lacking most of the first exon as a result of the gap in the genome (S5 Table).

There are also four annotated canine transcripts, one RefSeq and three Ensembl, which do not match any of the known human *KCNIP4* Ensembl transcripts (S5 Table). Two of these transcripts, ENSCAFT00000079461.1 and ENSCAFT00000061238.1, are indicated in the annotation to have an additional exon between exons 3 and 4. This additional exon is not seen in any of the human transcripts or any of the other annotated canine transcripts, and was not observed in canine cerebellum mRNA sequencing data. Ensembl transcript ENSCAFT00000026195.4 appears to be an incorrect amalgamation of the first exon of KCNIP4-1eΔ2 (XM_005618660.3), the second exon of KCNIP4-1a, and exons 3 to 9 which are present in all of the *KCNIP4* transcripts (Fig 1A). This transcript was not seen in the mRNA sequencing data. Canine RefSeq transcript XM_536275.6 is stated to start with the second exon of KCNIP4-1a, followed by exons 3 to 9. This combination of exons is not seen in any of the annotated human Ensembl transcripts.

RT-PCR of RNA extracted from cerebellum tissue samples from two ataxia-affected Buhunds and five unaffected dogs of other breeds confirmed that at least five transcripts for *KCNIP4*, with alternative first exons, are expressed in the canine cerebellum (Fig 1B). The variant identified in this study is located in exon 6, which Sanger sequencing of the RT-PCR products for the Buhund siblings and a Golden Retriever confirmed is expressed in all five of these transcripts (Fig 1C). All of the confirmed transcripts contain exons 3 to 9.

### RT-qPCR of KCNIP4 expression

The relative expression of *KCNIP4* in cerebellar tissue samples from the two sibling cases and five unaffected dogs, in comparison to the ubiquitously expressed TATA box binding protein (*TBP*) gene, was assessed using RT-qPCR. The assay for *KCNIP4* was designed with primers in exons 3 and 4, both of which are present in all five of the transcripts shown to be expressed in the canine cerebellum, allowing the assay to quantify total *KCNIP4* transcript expression.

**Fig 2:**
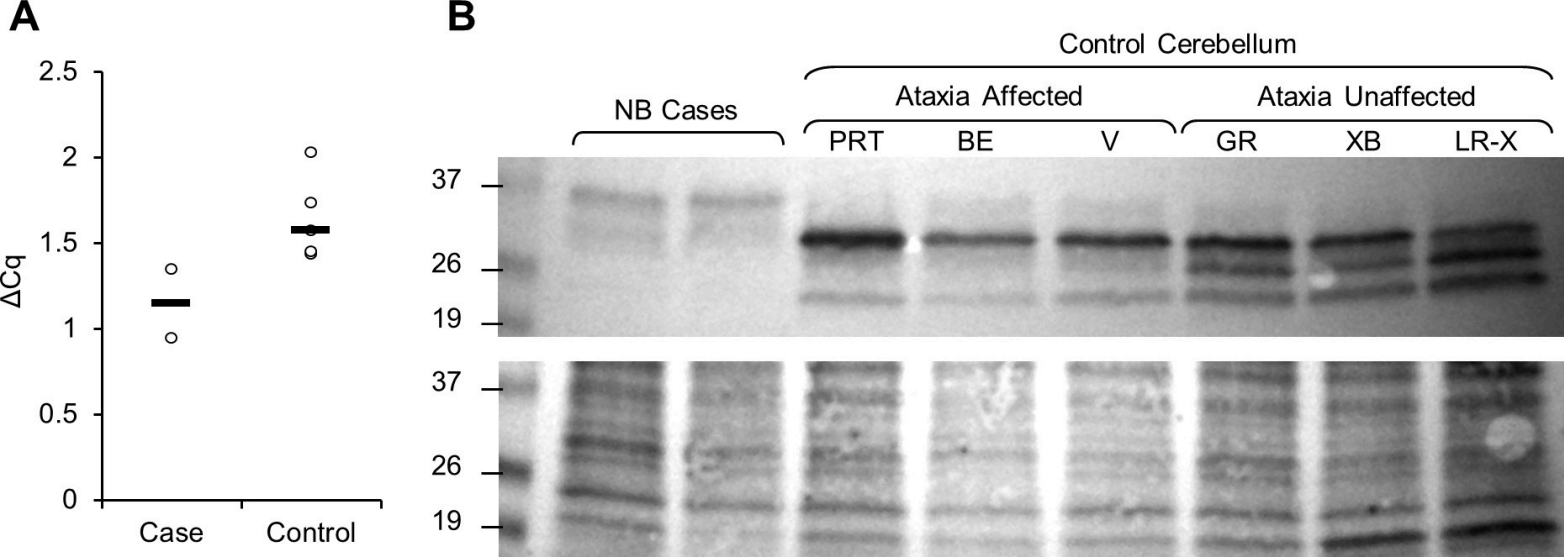
(A) Relative quantification (ΔCq) of *KCNIP4* in comparison to the ubiquitously expressed control gene *TBP* in cerebellar tissue samples of two ataxia affected Buhund siblings (Case) and five ataxia unaffected dogs (Labrador Retriever cross-breed, Siberian Husky cross-breed, Beagle, Golden Retriever, and a Great Dane) (Control). Black lines show group median. (B) Western blot comparing KCNIP4 protein expression in the cerebellum tissue lysate of two Norwegian Buhund cases and six control dogs. Top panel: Anti-KCNIP4 western blot. Bottom panel: Ponceau S total protein loading control. From left to right: two ataxia affected Norwegian Buhunds (NB); Three ataxia-affected control dogs of other breeds (Parson Russel Terrier (PRT); Beagle (BE); Hungarian Vizsla (V)); three ataxia-unaffected control dogs of other breeds (Golden Retriever (GR); Siberian Husky cross breed (XB); Labrador retriever cross breed (LR-X)). Approximate sizes of protein ladder bands are indicated on the left (kDa).

Relative quantification of *KCNIP4* was suggestive of reduced expression in cerebellar tissue from the two affected dogs (ΔΔCq = 0.497) (Fig 2A), although the change in expression was not statistically significant (Student’s T-test, p = 0.07).

### Western blot shows loss of KCNIP4 in cerebellum of cases

Western blot analysis was carried out for KCNIP4 using cerebellum tissue lysate from two cases and six control dogs. Three of the controls were affected by cerebellar ataxia but were of other breeds for which the causal mutation is known, and the remaining three were unaffected by ataxia. The western blot showed a clear change in KCNIP4 expression in the cerebellum tissue from the Buhund case (Fig 2B). Three bands are visible for the control tissues, at approximately 28 kDa, 26 kDa, and 22 kDa. The band with the highest saturation in all controls is at 28 kDa, with the lower molecular weight bands observed to be fainter in the three controls which were affected by genetically distinct forms of cerebellar ataxia. The two lower bands are absent, or too faint to observe, in the two Buhund cases. The 28 kDa band appears to still be present in the Buhund cases, but at a much lower saturation. A fourth band is observable for all eight tissue lysates at approximately 37 kDa, and has a higher saturation in the two Buhund cases. The different bands may represent the differently sized isoforms of KCNIP4, although the band at 37 kDa does not fit within the size ranges of any of the known KCNIP4 isoforms (S5 Table). The western blot analysis indicates a dramatic reduction of KCNIP4 expression in dogs homozygous for the *KCNIP4* variant, and could suggest a complete loss of some, if not all, KCNIP4 isoforms.

### Immunohistochemistry shows a reduction of KCNIP4 expression in the cerebellum of an affected Buhund

**Fig 3:**
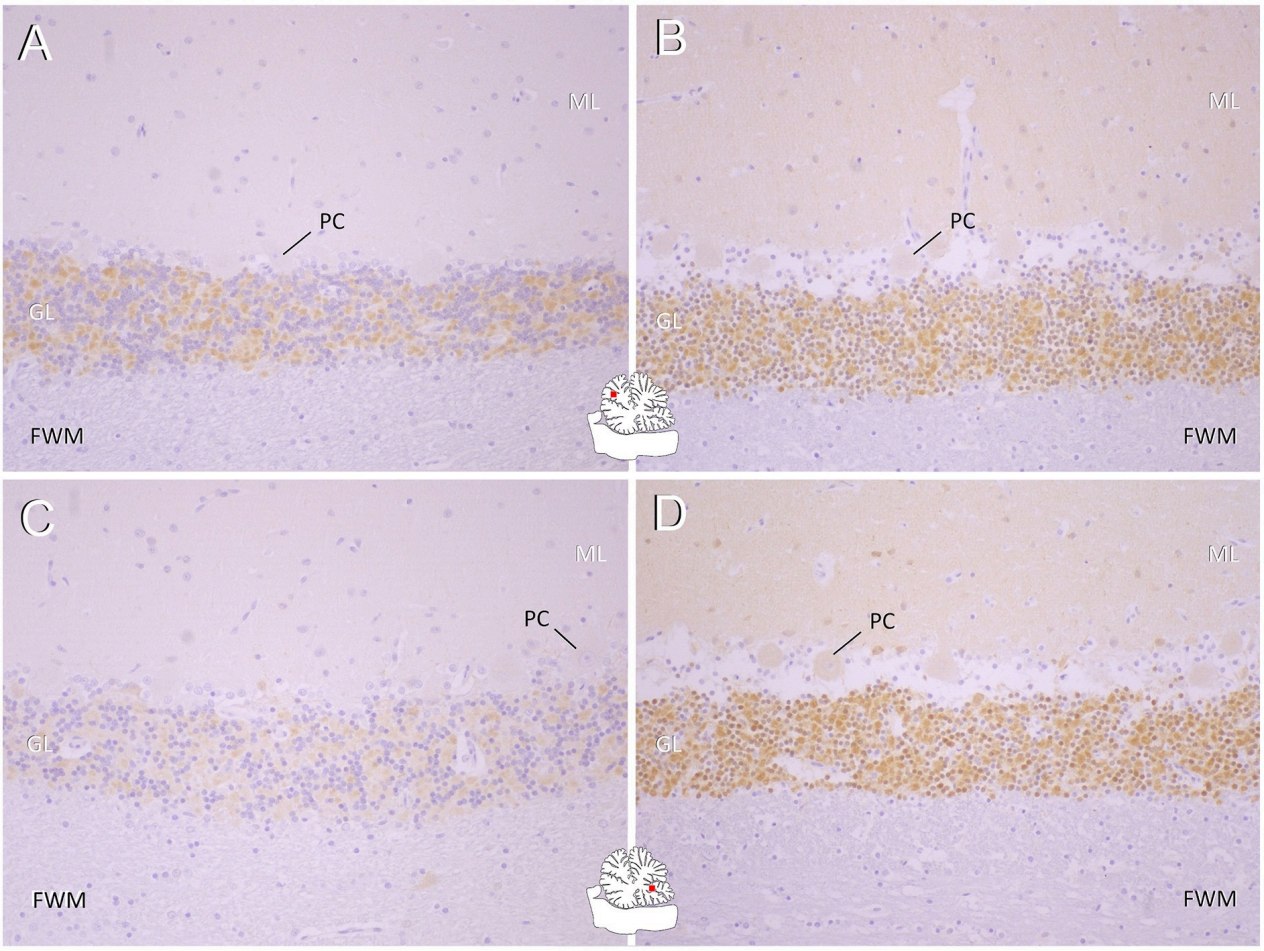
Immunohistochemical expression patterns of KCNIP4 in the cerebellum of an affected Buhund (A, C) compared to that of two control dogs (B,D). Top panels: Rostral lobe. Bottom panels: Caudoventral vermis. The Buhund shows a diffusely decreased immunopositivity within the molecular layer (ML) and granular layer (GL) (brown staining). Thereby, rostral lobe areas (A) present with slightly stronger signal intensity than the caudoventral vermis (C). This difference matches to regional differences in Purkinje cell marker expression demonstrated previously [18]. PC: Purkinje cell; FWM: foliary white matter.

Expression of KCNIP4 protein was identified immunohistochemically throughout synaptic glomeruli of the cerebellar granular cell layer and basket cells of the molecular layer in all dogs, but staining intensity was considerably lower in the Buhund with two copies of the mutation (Fig 3). As functional differentiation of Purkinje cells appears to underlie regional differences [18], sagittal and transverse sections of all cerebellar lobuli were examined. Reduction of KCNIP4 expression was seen throughout the entire cerebellar cortex but more extensive in areas where Purkinje cells showed least expression of calbindin and ITPR-1 in the histopathological analysis carried out previously for the clinical characterisation of the disease [18].

### In Silico protein analysis of the 3D structure of KCNIP4 suggests that the mutation affects protein stability and function

The structure of one KCNIP4 isoform, KChIP4a (KChIP4 was a previous abbreviation for KCNIP4), has been determined through X-Ray diffraction (PDB ID 3DD4). KChIP4a aligns perfectly with XP_003434448.1 and KCNIP4-204 (KCNIP4-1dΔ2). This allowed the use of online tools for predicting the effect of the variant on protein stability. The Eris server, which uses discrete molecular dynamics, was used to predict the effect of the mutation on the free energy of the protein’s structure [24]. The tryptophan to arginine substitution caused a predicted ΔΔG of 7.31 kcal/mol, which indicates a dramatic decrease in stability.

**Fig 4:**
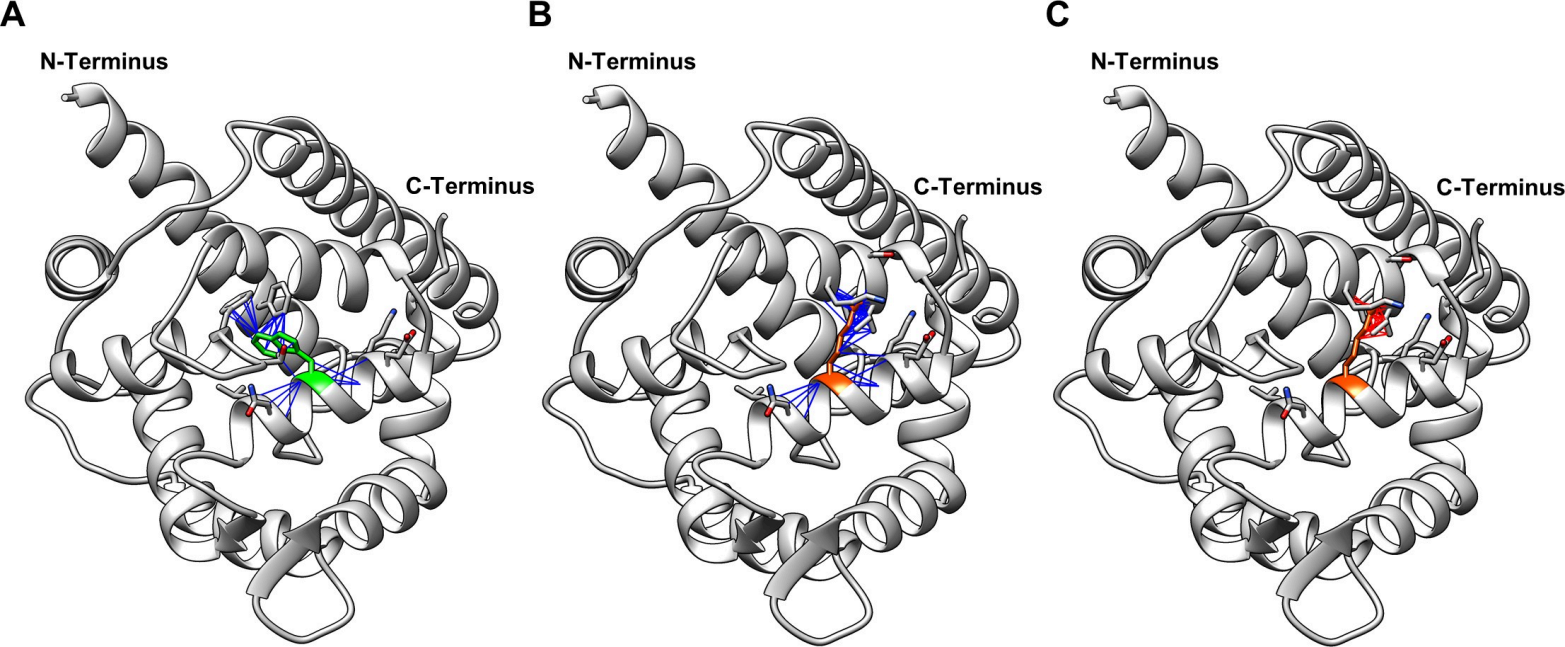
3D models of KCNIP4 with and without the amino acid substitution caused by the mutation identified. (A) KCNIP4 with tryptophan (green) at position 142. (B) KCNIP4 with arginine (orange) at position 142. Blue lines in A) and B) indicate predicted interactions between atoms (including polar and nonpolar interactions). (C) KCNIP4 with arginine (orange) at position 142, with red lines indicating clashes (interactions where atoms are too close together).

The 3D protein structure was used to investigate the physical location of the amino acid substitution within the protein. The tryptophan residue affected is within the hydrophobic core of KCNIP4, and is predicted to interact with the N-terminal helix which sits within the groove of the protein (Fig 4A). The substitution replaces the non-charged, non-polar, hydrophobic tryptophan with the positively charged, polar, arginine. This is likely to have an impact on the hydrophobicity of the protein’s core. The arginine residue is predicted to interact with different helical structures within the protein compared to tryptophan, no longer interacting with the N-terminal helix (Fig 4B). The arginine residue is also predicted to overlap, and clash, with neighbouring residues (Fig 4C).

## Discussion

In the present study we used whole genome sequencing to identify the causal mutation for a recently characterised cerebellar ataxia in the Norwegian Buhund dog breed [18]. Whole genome sequencing of single cases of ataxia has recently been used to successfully identify mutations causing cerebellar cortical degeneration (CCD) in the Hungarian Vizsla and spinocerebellar ataxia in Russell Group Terriers [5, 17]. Taken together this suggests that cerebellar ataxias in the dog are particularly amenable to this approach for the identification of causal mutations. The approaches used in these two previous studies, however, used the candidacy of the genes in which variants were found to filter for potentially causal variants [5, 17]. The present study used a much larger number of in-house and consortium control sequences to filter variants to a level manageable for follow-up, allowing the discovery of a likely-pathogenic variant in *KCNIP4*, a novel gene for cerebellar ataxia. A previous study that used GWAS and targeted resequencing to investigate a different form of canine cerebellar ataxia in Russell Terrier Group dogs identified a variant in *CAPN1*, which had also not previously been implicated in ataxia in humans [15]. Mutations in this gene were later demonstrated to cause autosomal-recessive hereditary spastic paraplegia in humans, which is a condition that can present with cerebellar signs and which is associated with ataxia in some cases [25]. Canine hereditary cerebellar ataxia, as a naturally occurring disease model, therefore represents a resource for the identification of novel genes that should be considered as potential candidates when investigating similar conditions in humans. The discovery of novel genes for hereditary ataxia (and other rare, autosomal recessive, or autosomal dominant diseases) in the dog, when only very small numbers of cases are available, is therefore becoming increasingly possible with the rapid expansion of publicly available whole genome sequence datasets [19]. In the future this could make clinical diagnostic sequencing affordable and efficient for emerging Mendelian canine conditions.

The mutation identified is a nonsynonymous SNP causing a tryptophan to arginine amino acid substitution within a highly conserved region of KCNIP4. Voltage-gated potassium channel-interacting proteins (KCNIP, previously called KChIP) are four calcium binding proteins which interact with voltage-gated potassium (Kv) channels and modulate A-type potassium currents [26, 27]. Mutations in *KCNIP4* have not been associated with cerebellar ataxia previously in any species to the authors’ knowledge. However, mutations within *KCND3*, which encodes the voltage-gated potassium channel α-subunit Kv4.3, have been found to cause spinocerebellar ataxia type 19/22 in humans [28–32]. Mutations in *KCND3* reduce trafficking and cell surface expression of KV4.3 and suppress the amplitude of the potassium current or affect channel gating [33]. KChIP4a, an isoform of KCNIP4, has been shown to interact with Kv4.3 and to modulate its inactivation [34, 35].

RT-qPCR analysis did not demonstrate a statistically significant reduction in the expression of the *KCNIP4* transcript in the affected Buhunds, although it was suggestive of reduced expression; whereas a dramatic reduction in KCNIP4 protein expression was observed in the cerebellum of cerebellar ataxia-affected Norwegian Buhunds, through both western blot and immunohistochemistry. It has been demonstrated previously that changes in the protein expression of the Kv4 accessory subunits (KCNIP1, KCNIP2, KCNIP3, and KCNIP4) do not necessarily reflect transcriptional expression [36]. It has been suggested that the formation of complexes between the accessory subunits and the α-subunits (Kv4.2 or Kv4.3) stabilises the proteins, leading to increased levels of each protein [36]. The tryptophan to arginine substitution identified in the current study is predicted to be destabilising and damaging to protein function. If the predicted effect on protein function prevents the formation of complexes, and this precludes the stabilisation of the protein, combined with the mutation’s destabilising effect this could potentially explain the dramatic reduction in protein expression observed without a significant drop in transcript expression. Only RNA from two cases was available for the RT-qPCR analysis; inclusion of a larger number of cases would be necessary to confirm this finding.

Although the western blot and immunohistochemical analysis indicate a considerable reduction in KCNIP4 protein expression in affected dogs, the exact epitope of the antibody used is unknown. The immunogen used by the manufacturer consisted of the majority of the protein’s amino acid sequence, and the epitope has not been mapped. This means that the epitope could be in the region of the mutation, and the mutation could potentially have prevented antibody binding and thus mimicked an apparent fall in protein expression. However, the immunohistochemistry demonstrated that there was still some expression in a case, and that the expression pattern matched that seen previously for KCNIP4 expression in humans and mice [23], and the variability in expression in the case match that of the Purkinje cell markers demonstrated in the histopathological analysis carried out previously [18]. This indicates that the antibody used is still capable of binding the protein containing the variant.

An additional band, at a greater atomic mass than the known KCNIP4 isoforms, was observed on the western blot. This additional band with a mass of ~37 kDa, which had a higher intensity in the two cases, could potentially represent an increase in expression of a different accessory Kv4 subunit, observable as a result of cross-reactivity of the antibody used. Loss of one of the KCNIPs has previously been observed to result in an increase in protein expression of the other, non-disrupted KCNIPs [36]. This compensatory mechanism is thought to occur through competition between the KCNIPs for binding to α-subunits [36, 37]. The stabilisation that occurs through binding to the other subunits results in an increase in protein levels.

The three main bands are observed for the control tissues in the western blot, at approximately 28 kDa, 26 kDa, and 22 kDa, are expected to represent the various isoforms of KCNIP4, which fit within this approximate size range (S5 Table). The band with the highest saturation in all controls, at 28 kDa, has a size closest to that expected of KCNIP4-1a. The lower two bands were observed to be fainter in the three controls which were affected by other forms of cerebellar ataxia, in comparison to those unaffected by ataxia. We propose that this difference could be a result of cerebellar degeneration, particularly for the Beagle and Hungarian Vizsla CCD cases, which are forms of ataxia characterised by the loss of Purkinje cells and depletion of the granular cell layer [5, 16]. The degradation of the cerebellum and cell types known to express the KCNIP4 protein could feasibly have resulted in the reduction in KCNIP4 expression observed in comparison to the unaffected controls. In comparison, the Norwegian Buhund cerebellar ataxia cases, which had two copies of the *KCNIP4* variant but negligible cerebellar degeneration observed in the histopathological analysis [18], demonstrated almost complete loss of KCNIP4 protein expression in the western blot analysis, and dramatically reduced expression in the immunohistochemistry, indicating that in these individuals the loss of protein is caused by the mutation, not the cerebellar degeneration.

Human *KCNIP* genes have seven 3’ exons (exons 3 to 9, Fig 1A) which are identical in length and have highly similar sequences between the four genes [23]. The 5’ exons for the four genes are extremely dissimilar, and each *KCNIP* gene has multiple 5’ exons which are unique. The high homology of the genes indicates shared protein function, and the conservation of the amino acid sequence of exons 3 to 9 suggests that variants in these regions are likely to be damaging to function. The variant identified in this study is located within exon 6, one of the highly conserved 3’exons, in a region of the protein which has identical amino acid sequences in the four canine proteins. The bioinformatics tools we have used show that the mutation in this conserved region affects the hydrophobic core of the protein, and predict it to be destabilising and damaging to function.

In addition to demonstrating that a variant within *KCNIP4* causes cerebellar ataxia in the Norwegian Buhund dog breed, we have characterised the gene’s expression in the canine cerebellum. RT-PCR analysis and in situ hybridisation studies have demonstrated that *KCNIP4* is expressed in the cerebellum in humans and mice [23]. We have demonstrated that at least five *KCNIP4* transcripts are expressed in canine cerebellum, and that they share high sequence similarity with the human transcripts, all sharing the seven highly conserved 3’ exons but with alternative 5’ exons aligning with those seen in humans. A previous *in situ* hybridisation study in mice demonstrated that *KCNIP4* is expressed in the Purkinje cell layer and granular layer neurons of the cerebellum [23], and a immunohistochemical study in the rat showed protein expression throughout the cerebellum but particularly in the granular cell layer [38]. We demonstrate that KCNIP4 protein is also expressed strongly in the granular layer of the canine cerebellum. One transcript was only found in the kidney in humans [23]. Consistent with this, this transcript, KCNIP4-1cΔ2, was not identified in the canine cerebellum. However, the first exon of this transcript is a shortened version of the first of the 3’ exons shared by all transcripts, which makes it impossible to assay using the methods used here; primers designed for this first exon would amplify all KCNIP4 isoforms. We were unable to locate the 5’ untranslated region (UTR) for the KCNIP4-1cΔ2 transcript in canine cerebellum mRNA sequencing data.

In the immunohistochemical analysis we observed that expression of KCNIP4 in the canine cerebellum was predominantly within the granular cell layer, and KCNIP4 expression was strong throughout the synaptic glomeruli. Previous research has suggested a role for A-type potassium channels in the regulation of postsynaptic excitability of granule cell dendrites at the synapse between granule cells and mossy-fibre cells [38]. We suggest that a theoretical effect of the mutation described in the present study could therefore be hyperexcitability of postsynaptic granule cell membranes and/or receptor potentials being lost in the dendritic tree instead of travelling to granule cell soma and via parallel fibres to Purkinje cells. We theorise that the features observed in Purkinje cells [18] could be secondary to uncoordinated signalling from granule cells. Interestingly, an *in situ* hybridisation study in mice demonstrated that the *KCNIPs* that are expressed in the cerebellum, including *KCNIP4*, are expressed at a higher level in the rostral lobe when compared to the caudal vermis [23]. Our immunohistochemical findings showed the expression of KCNIP4 protein was lowest in the caudoventral vermis of cases when compared to the rostral lobe, and this is also the region where the previous histopathological findings showed the least expression of Purkinje cell differentiation marker proteins (calbindin and ITPR-1) [18].

A potential limitation of the present study is the small number of affected dogs included in the research; increasing the risk of a false positive finding. This is a result of the numerically small size of the Buhund breed, and the limited availability of samples from affected dogs. However, the availability of obligate carriers and other individuals from the extended pedigree moderates this as a limitation. Genotyping large numbers of unaffected breed matched controls, and multiple representatives of many different breeds, also minimised the effect of this limitation. We were also unable to breed-match when performing RT-qPCR, western blot and immunohistochemistry due to the lack of availability of relevant tissue, but this limitation was minimised by the use of control samples from dogs of 3–6 diverse breeds. A potential limitation of the WGS analysis pipeline used here is that it would not have detected structural variants or insertions and deletions larger than approximately 75 bp. Given that we identified a compelling candidate causal variant in our current pipeline, we did not further investigate larger structural or non-coding variants. However, both would require the use of gene candidacy to generate a realistic number of variants for follow-up. Future refinement of structural variant callers will enable their incorporation into pipelines for efficient analysis of WGS, with necessary harmonisation amongst datasets.

In summary, by whole genome sequencing two Norwegian Buhund siblings, and filtering against a bank of genomes of dogs of other breeds, we have identified a mutation associated with cerebellar ataxia in this breed. The mutation is in a gene, *KCNIP4*, not previously implicated in this disease in any species, and these findings could therefore inform research into inherited ataxia of unknown aetiology in humans. This research has led to the development of a DNA test for cerebellar ataxia in the Norwegian Buhund breed, allowing dog breeders to avoid producing affected dogs, reduce the allele frequency, and eventually eliminate the disease from the breed.

## Methods

### Ethics statement

Samples were collected from privately owned pet dogs from the general population, and samples from the UK were in the form of buccal swabs or residual blood samples taken as part of a veterinary procedure. This study was approved by the Animal Health Trust ethics committee (AHT06-09). EDTA blood samples (3 ml) from 40 Norwegian Buhunds donated to research were collected under the permission of animal ethical committee of County Administrative Board of Southern Finland (ESAVI/343/04.10.07/2016) and all experiments were performed in accordance with relevant guidelines and regulations and with owners’ written consent.

### Sample collection and DNA extraction

Samples from four Norwegian Buhunds diagnosed with cerebellar ataxia by veterinary neurologists at the Animal Health Trust Centre for Small Animal Studies, Newmarket, were included in this research. Buccal swabs and post mortem tissue samples (stored in RNAlater) were collected from two affected siblings diagnosed in 2008 (aged 12 weeks (0.2 years)). For the dogs diagnosed in 1998 and 2002 (aged 16 and 20 weeks, respectively (0.3 and 0.4 years)) residual formalin-fixed paraffin-embedded (FFPE) tissue samples, collected for histopathology at the time of diagnosis, were used. Clinical descriptions and details of diagnosis have been described previously [18].

For validation of potential causal variants, samples from three sets of unaffected Norwegian Buhunds were utilised. The first set of DNA samples from 70 UK dogs were archived samples which had previously been collected for an unrelated study between 2008 and 2011, with the exception of one dog sampled in 2015 (“UK Set 1”). The dogs in this set were aged between 0.3 and 15.3 years (mean age 5.5 years) at sample collection, and none had been reported to have ataxia by their owners. Two sets of DNA samples were collected in 2017 from Norwegian Buhunds reported to have no signs of ataxia; one set from 36 dogs in the UK (“UK Set 2”) and another from 40 dogs living in Finland (“Finnish Buhund Set”). Dogs in “UK Set 2” were aged 0.9 to 8.8 years (mean age 4.2 years), and in the Finnish Buhund Set were aged between 0.6 and 9.5 years (mean age 3.2 years).

DNA was extracted from buccal swabs using the QIAamp Midi Kit (Qiagen), or whole blood using a standard chloroform protocol. DNA extraction from FFPE samples was carried out using Recoverall total nucleic acid isolation kit (Ambion). In the Finnish cohort, genomic DNA was extracted from the white blood cells using a semi-automated Chemagen extraction robot (PerkinElmer Chemagen Technologie GmbH, Baeswieler, Germany) according to the manufacturer’s instructions. DNA concentrations were measured using Qubit fluorometer (Thermo Fisher Scientific, Waltham, Massachusetts, USA) and Nanodrop ND-1000 UV/Vis Spectrophotometer (Nanodrop technologies, Wilmington, Delaware, USA) and samples were stored at –20°C.

### RNA sequencing

Genome-wide RNA sequence analysis was carried out, using extant data generated as described previously [18]. Visual inspection of gene transcripts was carried out using the Integrative Genomics Viewer (IGV) [39].

### Whole genome sequencing

Whole genome sequencing, including library preparation, of the two affected Norwegian Buhund siblings was carried out by Edinburgh Genomics laboratories, University of Edinburgh, using Illumina 150 bp paired-end sequencing (approximately 40X coverage). The library preparation method used was TruSeq DNA Nano (Illumina). Sequence reads were aligned to the canine reference genome CanFam3.1 using the Burrows-Wheeler Aligner (BWA-MEM), and SNP and in-del variants were called using the Genome Analysis Toolkit (GATK) Haplotypecaller (v3.6) using GATK best practices [40, 41]. Consequence predictions were designated for each variant using the Variant Effect Predictor (Ensembl), and variant calls for genomes of 44 unrelated dogs of 29 other breeds, were used to filter variants [42]. The initial 44 control genomes, and the additional 140 that later became available for assessment of candidate variants, had been accrued over time for other research and as a resource.

### Genotyping

The initial genotyping for candidate variants identified as potentially causal was performed using Sanger sequencing for eight SNPs, and fragment length analysis for a deletion, on an ABI 3130XL Genetic Analyzer. Primer sequences are available in S6 Table. Hotstartaq plus (Qiagen) was used for the initial PCR prior to sequencing, using the following cycling conditions: 95°C for 5 minutes, followed by 35 cycles of 30 seconds each at 95°C and 57°C, and 72°C. Sanger sequencing used Bigdye terminator v3.1 ready reaction mix (Applied Biosystems), using standard cycling conditions: 96°C for 30 seconds, followed by 44 cycles of 4 seconds at 92°C, 4 seconds at 55°C and 1 minute 30 seconds at 60°C. Fragment length analysis was carried out using a three primer system, utilising a forward primer designed with a “tail” at the 5’ end and a fluorescently labelled (FAM) primer complementary to the “tail” on the forward primer [43]. Hotstartaq plus (Qiagen) was used for PCR, and cycling conditions were as follows: 94°C for 4 minutes, followed by 30 cycles of 30 seconds each at 94°C and 57°C, and 1 minute at 72°C. This was followed by eight cycles of 30 seconds each at 94°C and 50°C, and 1 minute at 72°C. The cycling was concluded with a 30 minute extension step at 72°C.

Further genotyping for the *KCNIP4* variant utilised an allelic discrimination method, using an ABI StepOne real-time thermal cycler. The assay primer and probe sequences are in S7 Table. The Kapa probe fast master mix (Kapa Biosystems) was used for genotyping all dogs, except those for which samples were collected as FFPE tissue. To optimise genotyping of the latter by limiting the levels of PCR inhibitors, DNA isolated from FFPE samples was further purified by ethanol precipitation before genotyping. FFPE derived DNA samples were genotyped using the Taqpath Proamp master mix (Applied Biosystems). For allelic discrimination assays using KAPA Probe Fast and DNA from buccal swabs or blood samples, a fast ramping speed was used and the following cycling conditions were used: 30 seconds Pre-PCR read at 25°C, 95°C holding stage for 3 minutes, 40 cycles of 95°C for 3 seconds and 60°C for 10 seconds, followed by a 30 second post-PCR read step at 25°C. Allelic discrimination assays using Taqpath Proamp used the standard ramping speed and the following cycling conditions: 60°C for 30 seconds pre-PCR read, 5 minute 95°C holding stage, 40 cycles of 15 seconds at 95°C and 1 minute at 60°C, and 60°C for 30 seconds post-PCR read step.

### RT-qPCR

RNA was extracted from cerebellum samples collected post-mortem from the two affected Norwegian Buhund siblings and five other dogs unaffected by ataxia of different breeds (Labrador Retriever cross-breed, Siberian Husky cross-breed, Beagle, Golden Retriever, and a Great Dane) Elimination of genomic DNA and reverse transcription was carried out using the Quantitect cDNA synthesis kit (Qiagen). Assay primers and probe were designed for qPCR with the fluorescently labelled probe overlapping the boundaries of exons present in all alternative *KCNIP4* transcripts (Forward: GCCCAGAGCAAATTTACCAAG, probe: AAGAATGAGTGTCCCAGCGGTGT, reverse: CGGAAAGAACTGCGAGTAAATC). The qPCR was carried out using Luna Universal qPCR Master Mix (NEB) and an ABI StepOnePlus real-time PCR system, and comparative CT analysis used for relative quantification compared to an assay for the ubiquitously expressed TATA box binding protein (*TBP*) gene. Reaction efficiency was calculated by performing a seven point, doubling dilution, standard curve. The reaction efficiencies of both the *KCNIP4* and *TBP* assays were 99.2%.

### PCR and sanger sequencing of transcripts

PCR primers were designed to be specific to each transcript, with a unique forward primer designed to overlap the boundary of the first and second exon and a reverse primer in the final exon which all transcripts share: GCATGGAGCGCATTATGTTT (S8 Table). Hotstartaq plus (Qiagen) was used for PCR of the cDNA produced by reverse transcription for the two Buhunds and five control dogs (see above), and cycling conditions were as follows: 95°C for 5 minutes, followed by 35 cycles of 30 seconds each at 95°C and 57°C, and 72°C. This was followed by final step at 72°C for 5 minutes. Agarose gel electrophoresis (1.5% agarose, 100v for one hour) was carried out using 3μL of each product and images were taken. Sanger sequencing was carried out on the products for the two Norwegian Buhunds and Golden Retriever, as described above.

### Western blot

Cerebellum tissue from the two cerebellar ataxia-affected Norwegian Buhund siblings and six control dogs were homogenised in radioimmunoprecipitation assay (RIPA) buffer (Cell Signalling Technology). The six control dogs included three dogs affected by cerebellar ataxia, but clear of the *KCNIP4* mutation and of breeds for which the causal mutation is known (one of each of Parson Russel Terrier, Beagle, and Hungarian Vizsla). The Parson Russel Terrier was diagnosed with late-onset spinocerebellar ataxia, and both the Beagle and Hungarian Vizsla with CCD, and all three were homozygous for the applicable causal variant for their form of ataxia [5, 15, 16]. The remaining three control dogs were unaffected by ataxia and clear of the mutation: one of each of Golder Retriever, Siberian Husky cross breed, Labrador Retriever cross-breed. Total protein lysate (15 μl, 180–255 μg) was diluted in Laemmli sample buffer (BIO-RAD) and separated by SDS-polyacrylamide gel electrophoresis using a 4–20% Mini-PROTEAN TGX Stain-Free precast gel (BIO-RAD). Wet transfer was used to transfer proteins onto a nitrocellulose membrane (0.45 μm). Immunoblotting was carried out using a rabbit monoclonal primary antibody targeting KCNIP4 (abcam, ab203831). The immunogen for the primary antibody used was a recombinant protein covering all of KCNIP4 (KCNIP4-1a, ENST00000382152.7), from the first amino acid to the C-terminus. Protein band detection utilised the WesternBreeze anti-rabbit chromogenic kit (Invitrogen) which uses a goat anti-rabbit antibody and conjugated alkaline phosphatase for detection. Ponceau S stain was used as a total protein loading control.

### Immunohistochemistry

Tissue expression of KCNIP4 was evaluated immunohistochemically in transverse and sagittal sections of cerebella of one affected Buhund sibling and three neurologically healthy control dogs (Labrador Retriever, Australian Shepherd, Jack Russell Terrier). The staining employed the same antibody as used for western blot (as described above), polymer technology and a diaminobenzidine tetrahydrochloride detection kit. Slides were counterstained with haematoxylin and routinely coverslipped using xylene-based mounting medium.

### In silico protein analysis

*In silico* protein analysis was carried out using a 3D model (PDB ID 3DD4) obtained from the PDB-REDO Databank (pdb-redo.eu). The model had been refined and rebuilt from the original PDB model which had been determined through X-ray diffraction [35]. The model was visualised using UCSF Chimera software [44], and the two missing loops were modelled using Modeller [45]. Heteroatoms were removed from the PDB file, and a model of the protein containing the variant was created using Chimera’s Rotamers tool, choosing the arginine rotamer with the highest probability in the Dunbrack library [46]. Clashes and contacts were predicted using Chimera’s “Find Clashes/Contacts” tool which uses van der Waals radii to find interatomic clashes and contacts. The software’s default clash and contact criteria were used. Clashes: van der Waals overlap > = 0.6 angstroms, subtracting 0.4 for H-bonding pairs. Contacts: van der Waals overlap > = -0.4 angstroms.

## Supporting information

S1 Table Summary of the genotypes of 14 Buhunds for nine candidate variants. For two of the variants (*ITPR3* and ENSCAFG00000030891) one dog failed genotyping.
(XLSX)

S2 Table Genotypes of multi-breed panel by breed. (XLSX)

S3 Table The number of individuals for each breed represented in the 802 whole genome sequences used. (XLSX)

S4 Table UCSC Multiz Alignments of 100 Vertebrates Human GRCh38/hg38 Assembly 4:20,734,676–20,734,678. There are no alignment data for this region in the Gorilla.
(XLSX)

S5 Table Canine KCNIP4 transcripts and protein isoform annotations, and the details of those demonstrated to be expressed in canine cerebellum. (XLSX)

S6 Table Candidate causal variants identified from whole genome sequencing, and primer sequences used for Sanger sequencing and fragment length analysis. (XLSX)

S7 Table Primer sequences used for allelic discrimination assay of *KCNIP4* variant. (XLSX)

S8 Table Transcript-specific primers for *KCNIP4*. (XLSX)

S1 Fig Pedigree of affected dogs with obligate carriers and related dogs used for genotyping highlighted. (TIF)

S2 Fig UCSC Multiz Alignments of 20 species of mammal (17 primates) Human GRCh38/hg38 Assembly 4: 20,734,628–20,734,726. The amino acid at the location of the mutation is boxed, and the 16 flanking amino acids in each direction are shown. There are no alignment data for the Gorilla in this region.
(TIF)
