# Supplementary figures and images for: Characterisation of canine KCNIP4: A novel gene for cerebellar ataxia identified by whole-genome sequencing two affected Norwegian Buhund dogs
Source: PLoS Genet. 2020 Jan 30;16(1):e1008527. doi: 10.1371/journal.pgen.1008527 (PMC7012447; doi:10.1371/journal.pgen.1008527)

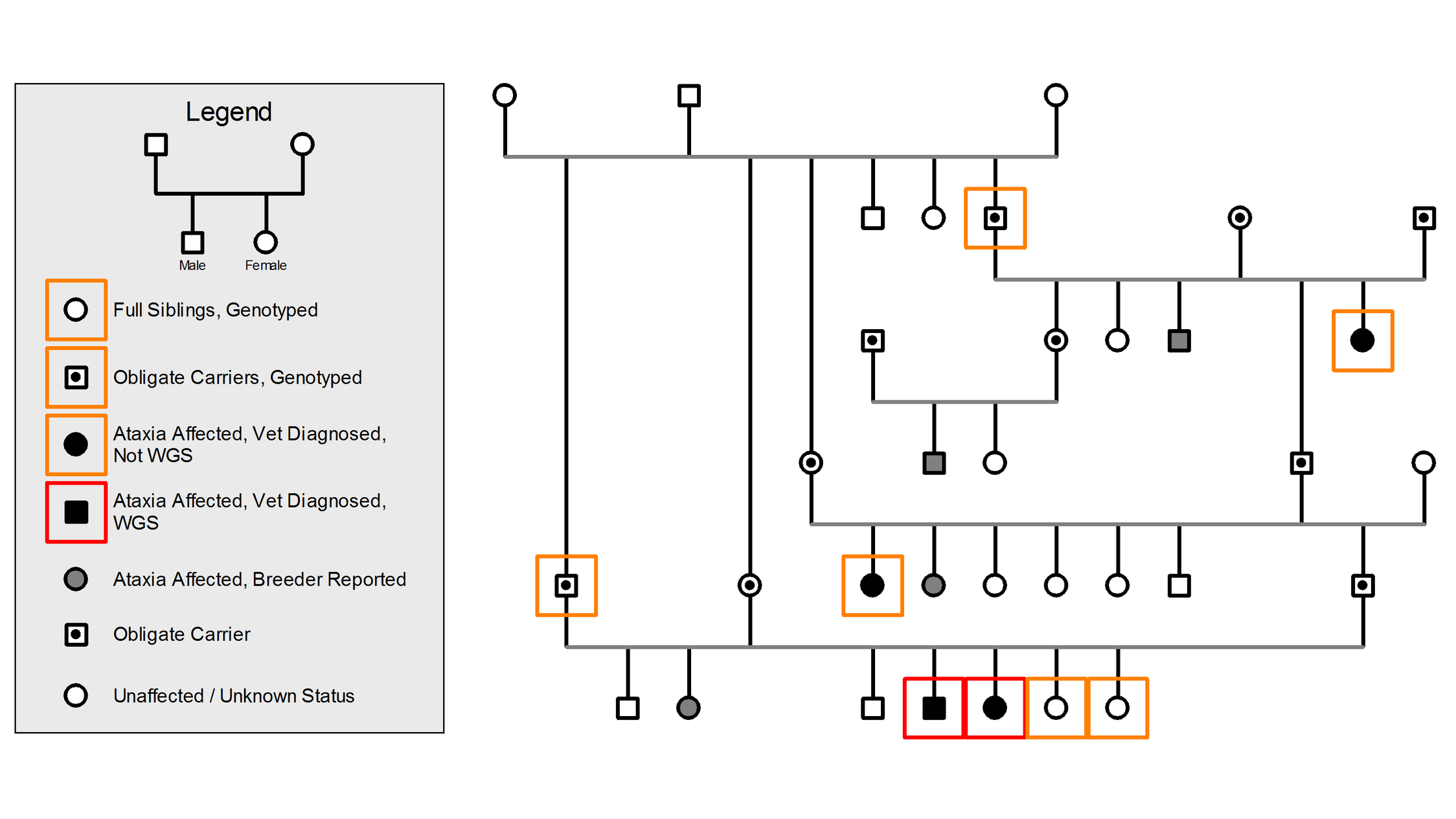

Supplement: S1 Fig — (TIF) [file pgen.1008527.s009.tif]

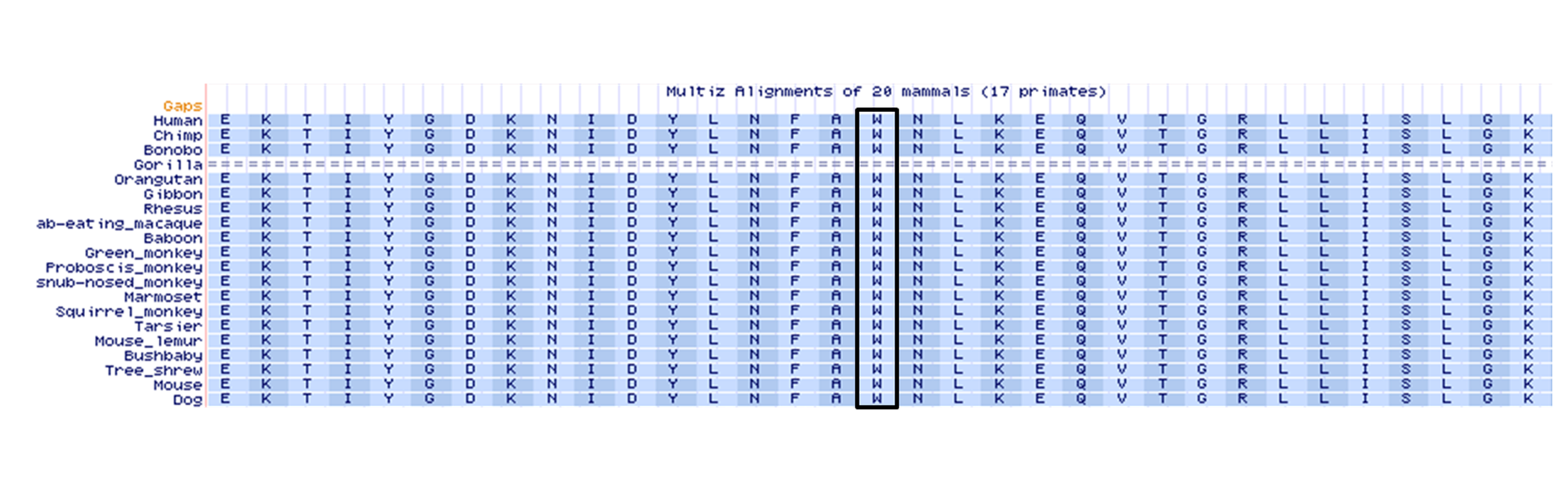

Supplement: S2 Fig — The amino acid at the location of the mutation is boxed, and the 16 flanking amino acids in each direction are shown. There are no alignment data for the Gorilla in this region. (TIF) [file pgen.1008527.s010.tif]
